# Research and Implementation of Travel Aids for Blind and Visually Impaired People

**DOI:** 10.3390/s25144518

**Published:** 2025-07-21

**Authors:** Jun Xu, Shilong Xu, Mingyu Ma, Jing Ma, Chuanlong Li

**Affiliations:** 1 School of Automation, Harbin University of Science and Technology, Harbin 150080, China; 19153303260@163.com (S.X.); 15546427528@163.com (M.M.); majing@hrbust.edu.cn (J.M.); lichuanlong@hrbust.edu.cn (C.L.); 2 Artificial Intelligence Robot Joint Laboratory, Harbin University of Science and Technology, Harbin 150080, China

**Keywords:** blind and visually impaired, deep learning, lightweight design, real-time performance, vibration feedback

## Abstract

Blind and visually impaired (BVI) people face significant challenges in perception, navigation, and safety during travel. Existing infrastructure (e.g., blind lanes) and traditional aids (e.g., walking sticks, basic audio feedback) provide limited flexibility and interactivity for complex environments. To solve this problem, we propose a real-time travel assistance system based on deep learning. The hardware comprises an NVIDIA Jetson Nano controller, an Intel D435i depth camera for environmental sensing, and SG90 servo motors for feedback. To address embedded device computational constraints, we developed a lightweight object detection and segmentation algorithm. Key innovations include a multi-scale attention feature extraction backbone, a dual-stream fusion module incorporating the Mamba architecture, and adaptive context-aware detection/segmentation heads. This design ensures high computational efficiency and real-time performance. The system workflow is as follows: (1) the D435i captures real-time environmental data; (2) the processor analyzes this data, converting obstacle distances and path deviations into electrical signals; (3) servo motors deliver vibratory feedback for guidance and alerts. Preliminary tests confirm that the system can effectively detect obstacles and correct path deviations in real time, suggesting its potential to assist BVI users. However, as this is a work in progress, comprehensive field trials with BVI participants are required to fully validate its efficacy.

## 1. Introduction

According to the Global Burden of Disease Study [1], in 2020, An estimated 43.3 million (95% UI 37.6–48.4) people worldwide were blind, with 55% (23.9 million) being female. Approximately 295 million (267–325) people had moderate and severe vision impairment, of whom 163 million (55%) were female. A total of 258 million (233–285) people had mild vision impairment, including 142 million (55%) females, while 510 million (371–667) people experienced visual impairment due to uncorrected presbyopia, with 280 million (55%) females affected. The study also reported global trends between 1990 and 2020 among adults aged ≥50 years. Age-standardized blindness prevalence decreased by −28.5% (95% UI −29.4 to −27.7). Mild vision impairment prevalence decreased slightly (−0.3% (−0.8 to −0.2)). Moderate and severe vision impairment prevalence increased (+2.5% (1.9 to 3.2)). Projections for 2050 based on this study [1] indicate that 61.0 million (52.9–69.3) people will be blind, 474 million (428–518) will have moderate and severe vision impairment, 360 million (322–400) will have mild vision impairment, and 866 million (629–1150) will have visual impairment from uncorrected presbyopia. The transportation and mobility problems of the blind and visually impaired (BVI), as an important livelihood issue that needs to be solved urgently, have long received extensive attention from the government and all sectors of society. Visual impairment poses great challenges to the blind and visually impaired in navigating complex environments and unfamiliar spaces, often triggering anxiety and fear. With the aging of the global population, the demand for visual aids is growing significantly as the group of blind and visually impaired people continues to expand. In order to alleviate the difficulties and high risks associated with mobility for the blind and visually impaired, visual guide technology is becoming an important aid.

The application of visual guidance technology involves a variety of environmental scenarios, including indoor, outdoor, and staircase, each of which faces different needs. In these complex environments, even if a blind or visually impaired person is equipped with a guide dog or a white cane to assist in traveling, it may still be difficult for him or her to walk safely due to the lack of timely and effective feedback. The information that blind and visually impaired people need in unfamiliar environments includes (1) environmental information such as road obstacles; (2) traffic guidance information such as crosswalks and blind alleys; and (3) orientation information such as one’s own position and direction. The travel risk of the blind and visually impaired can be effectively reduced only when this information is comprehensively and accurately transmitted. However, the existing market guide devices have a single function, and most of them only provide the obstacle avoidance function, which cannot meet the actual needs of the blind and visually impaired people in daily traveling [2]. At the same time, due to long-term wear and tear or irregular occupation of the blind road surface, there are serious spatial positioning problems, which increase the difficulty of the blind and visually impaired people walking [3]. At complex traffic intersections such as crosswalks and traffic lights, the travel risks for the blind and visually impaired are substantially greater. Their reliance on traditional guide canes, which lack real-time traffic feedback, leaves them at high risk of accidents in these busy environments.

To address the above problems, this paper proposes an innovative solution, an assistive device based on visual guide technology, combining deep learning algorithms and edge computing, aiming to explore a solution for providing comprehensive environment perception information for the blind and visually impaired in real time with the goal of helping BVI users avoid obstacles and navigate safely in future implementations. The main contributions of this paper are as follows:

- We present a portable and practical travel assistance device for the visually impaired. The system leverages the NVIDIA Jetson Nano for edge computing, integrates an Intel RealSense D435i depth camera for environment sensing, and utilizes an Arduino microcontroller with SG90 servos to provide intuitive vibration feedback, forming a robust hardware foundation for blind navigation.
- In response to the computational limitations of edge devices, we propose a novel, lightweight deep learning model for joint obstacle detection and walkable path segmentation. This model uniquely integrates a multi-scale attention mechanism with an efficient Mamba architecture and adaptive context-aware processing. Crucially, it achieves a special balance between high accuracy and real-time efficiency on embedded platforms.
- Extensive experimental validation demonstrates the superiority of our solution. Compared to state-of-the-art lightweight models (such as YOLOv9c-seg and YOLOv10n), our approach achieves exceptional accuracy in both tasks while maintaining an extremely compact model size (approximately 5MB) and high frame rates (>90 FPS), which are essential for real-time assistance for the visually impaired.

## 2. Related Work

Visually impaired people often rely on family members, canes, or guide dogs to navigate their environment. The most preferable method is to be accompanied by a family member; however, this approach does not improve the mobility independence of blind people. While canes provide some help, their ability to provide comprehensive environmental information is limited. Guide dogs, while more effective, face challenges in terms of popularity due to their high cost and complex training.

Consequently, contemporary guide devices have been developed, including electronic walking sticks, electronic guide dogs, and GPS guide systems. These devices employ technologies such as radar, infrared, and ultrasound to facilitate the perception of the surrounding environment and assist blind individuals in navigating their surroundings safely.

For example, Petsiuk et al. [4] proposed a low-cost, open-source ultrasound-based wearable bracelet type that conveys point-distance information by means of active detection, provides haptic feedback, and generates vibrations at different frequencies depending on the target range, but its narrow scanning angle and limited response speed make it easy to overlook the dangers posed by small, fast-moving objects. Our solution uses an RGB-D camera with a 90-degree horizontal field of view, which reduces blind spot coverage and, through a self-built dataset and detection algorithms, is able to identify common obstacles encountered by visually impaired individuals during travel, while also improving response speed. Papagianopoulos et al. [5] proposed a way to fix an infrared sensor on the top of a blind person’s arm to detect the internal environment of a building through the principle of infrared thermal imaging to guide the blind person to move, but it can only detect important obstacles in the blind person’s path, which has certain limitations. In contrast, our segmentation detection network can simultaneously segment drivable areas of regular roads, blind lanes, and crosswalks, and it can detect 12 types of obstacles in complex outdoor scenes, thus enhancing environmental perception capabilities.

With the continuous development of robotics, multiform and multifunctional guide robots continue to enter the public eye [6], avoiding dangerous accidents by sensing their surroundings and reducing the cognitive work required to navigate in unfamiliar areas. For example, Lu et al. [7] proposed an assisted guidance robot based on deep reinforcement learning (DRL) that can effectively avoid obstacles and achieve navigation in dynamic pedestrian environments. Although guide robots can help blind people move more independently, the development, manufacturing and maintenance costs of guide robots are high, and there are some technical challenges that make it difficult to be widely used. Our lightweight model does not rely on cloud training; instead, it can perform inference on edge devices, which results in lower power consumption and greater portability and is more suitable for outdoor use.

In recent years, with the rapid development of artificial intelligence technology, the GPU computing power continues to increase, leading to more applications of deep learning in the fields of image processing and environmental perception [8], and the convolutional neural network also occupies a place in the visual perception, so that the auxiliary travelling method based on deep learning has gained more attention from experts and scholars. Cao et al. [9] proposed a lightweight semantic segmentation network for the fast and accurate segmentation of blind lanes and sidewalks, using deep separable convolution and densely connected void space pyramid pooling modules to improve the segmentation speed and accuracy. However, there is a lack of obstacle detection work, and we have designed a unified multi-task model workflow that not only segments feasible areas but also detects obstacles during the walking process. Dimas et al. [10] proposed an obstacle detection method that combines deep learning, object recognition models, and human eye gaze prediction based on generative adversarial networks, aiming to provide outdoor navigation solutions for people with limited vision and conduct obstacle risk assessment and spatial localization through fuzzy logic methods to help users safely navigate and avoid obstacles. Ma et al. [11] proposed a blind obstacle segmentation system based on the E-BiSeNet neural network to help visually impaired people walk safely through real-time and accurate obstacle recognition and avoidance. This system only assisted the blind in obstacle avoidance, without providing guidance for a correct walking direction. In contrast, we timely corrected the walking direction of the blind by judging the angle of road deviation and the distance and angle between the blind person and obstacles. Hsieh et al. [12] proposed a wearable guidance device based on video streaming and deep learning to help blind or visually impaired people identify flat and safe walking routes by using RGB cameras and convolutional neural networks (CNNs) to convert images into depth maps. We used a stereo depth camera, which has a smaller error compared to monocular depth estimation. Suman et al. [13] proposed a “Vision Navigator” framework for predictive analytics, which realizes real-time detection and classification of obstacles through intelligent folding canes and sensor shoes, combined with single detection (SSD) and recurrent neural network (RNN) models, and it improves the independent navigation ability of visually impaired people. It is necessary to operate the smart cane and wear sensor shoes at the same time, which creates high operational complexity. However, our device has a high level of integration, requiring no auxiliary tools; in its current prototype form, it can be worn for testing. Mai et al. [14] proposed an intelligent guidance system for the blind based on 2D LiDAR and RGB-D cameras that integrates laser SLAM and an improved YOLOv5 algorithm, which can identify a variety of obstacles in real time and guide intelligent crutches to avoid obstacles, providing efficient indoor and outdoor navigation functions for the visually impaired. The cost of the multi-sensor fusion scheme of LiDAR and RGB-D is relatively high; thus, we used a pure vision solution to reduce costs and improve cost-effectiveness. Chen et al. [15] proposed a wearable navigation device based on semantic vision SLAM and a mobile computing platform that extracts environmental semantic information in real time through RGB-D cameras and helps visually impaired people avoid obstacles and understand their surroundings in the form of voice feedback. Voice feedback can easily be interfered with by surrounding environmental sounds outdoors, while we use servo motor vibration feedback to provide blind people with a more realistic tactile perception, significantly improving their adaptation to outdoor noise.

Therefore, as a work in progress, this paper proposes a travel assistance system that integrates deep learning and edge computing, which uses the NVIDIA Jetson Nano main control platform and a variety of sensors, combined with lightweight and efficient road segmentation and obstacle detection algorithms, and it has an accurate drivable area segmentation and obstacle detection capabilities. Preliminary algorithmic and hardware integration results demonstrate feasibility, though full validation with BVI users remains ongoing.

## 3. Hardware System Build

NVIDIA Jetson nano is a small, cost-effective embedded platform designed for artificial intelligence (AI) and Internet of Things (IoT) applications, featuring a 128-core Maxwell-based GPU, a quad-core ARM Cortex-A57 processor, and 4GB of memory. It supports a wealth of development tools and AI frameworks and is suitable for scenarios such as robotics, intelligent monitoring, and IoT devices. Jetson Nano is easy to use, provides powerful computing power with the JetPack SDK, and has an active developer community, making it ideal for getting started and developing AI and embedded system applications. Table 1 shows the hardware configuration of deep learning models that can run deep learning models efficiently.

**Figure 1:**
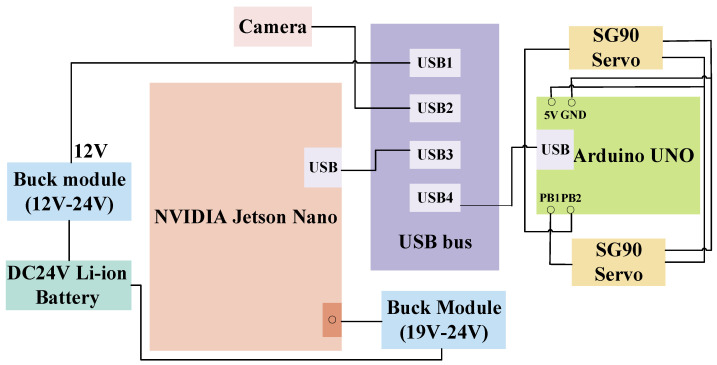
Hardware control system integration diagram.

The traveling aid for blind and visually impaired (BVI) people studied in this paper is mainly composed of NVIDIA Jetson nano, D435i depth camera, Arduino microcontroller, 2 SG90 servos, and a 24V lithium battery, and its hardware control system integration is shown in Figure 1.

**Figure 2:**
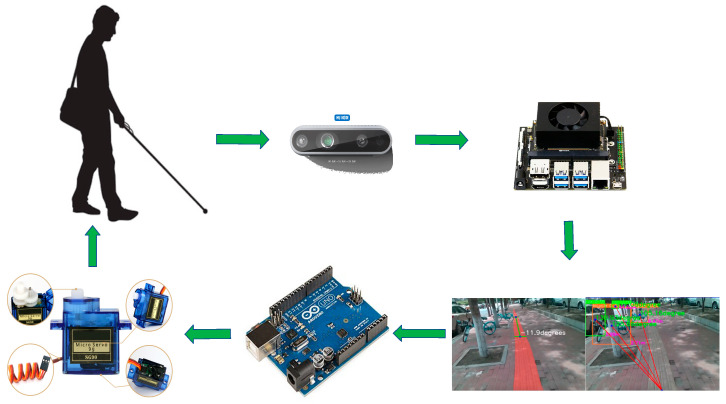
Device workflow diagram.

The overall flow of the equipment is shown in Figure 2. First, the depth camera D435i perceives the surrounding environment and depth information and then transmits the acquired image information to the AI edge computing device. After the lightweight detection and segmentation algorithm is processed, the corresponding offset and angle calculation are carried out on the detection and segmentation results, and the obstacle azimuth signal and road offset signal are generated by combining the depth information. The network output is sent to the Arduino microcontroller via the UART interface. The Arduino microcontroller connects two SG90 servos through GPIO pins, which vibrate below the person’s ears to alert the blind to adjust their walking path and avoid obstacles.

## 4. Method

**Figure 3:**
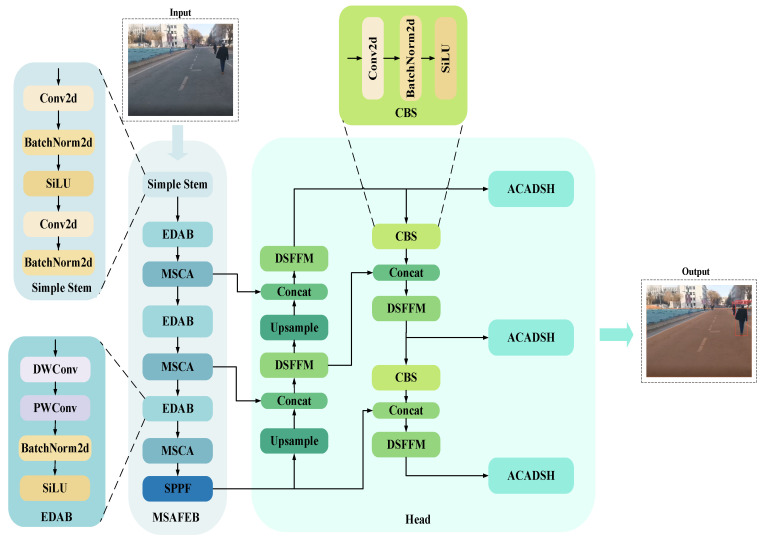
Diagram of the overall network structure.

In practical applications, although NVIDIA Jetson Nano provides good computational performance and supports the operation of common AI frameworks, due to its limited hardware resources (e.g., 128-core GPU and 4GB RAM), it is difficult to achieve high real-time requirements by directly running some complex deep learning models. Especially when dealing with larger-scale models or multitasking scenarios, the performance bottleneck of Jetson Nano leads to frame rate degradation, affecting the speed and accuracy of detection. Therefore, in order to achieve more efficient inference on embedded platforms, this paper designs a lightweight model to enhance its real-time and energy-efficient performance in resource-constrained environments. The network model structure is shown in Figure 3.

The network takes a 3-channel color image as the input and firstly passes through two convolutional layers with a step length of 2 and a kernel size of 3, combined with the activation function (SiLU) and normalization (BatchNorm, BN), and it performs preliminary feature extraction on the input image. This stage not only effectively extracts the basic features of the image, but it also lays the foundation for normalized and nonlinear expression for subsequent feature extraction. Subsequently, the network further improved the expression ability of features through the Efficient Depthwise Activation Block (EDAB) and the Multi-Scale Context Attention Unit (MSCA) stacked three times in a row. Among these, EDAB reduces the computational cost through deep separable convolution while preserving details; MSCA captures spatial and contextual information through multi-scale convolution and attention mechanisms, optimizes the selectivity of channels and spatial features, and significantly reduces redundant calculations. Finally, the features are fused at multiple scales through the SPPF module, which further enhances the unity of global semantics and detailed features.

In the decoding stage, the network uses stepwise up-sampling to recover the spatial resolution of features and combines the dual-stream feature fusion module (DSFFM) to realize the collaborative capture of information between global and local features. In the form of double branches, DSFFM focuses on local detail modeling and global context information extraction, which makes the feature expression in the decoding process richer. Subsequently, the fused features were further optimized by convolutional block search (CBS), and the CBS module strengthened the feature expression ability through convolution, activation, and normalization operations. At the same time, through multi-level up-sample and Concat operations, features at different levels are gradually fused, in which low-resolution global semantic features and high-resolution fine-grained features are organically combined, balancing the expression of details and semantic information and laying a solid foundation for detection and segmentation tasks.

Finally, the network completes the output of the final target detection frame and segmentation mask by combining grouped convolution, deformable convolutional network v4 (DCNv4), and adaptive context-aware detection and segmentation header (ACADSH) with channel attention mechanism. Grouped convolution reduces computational overhead, deformable convolution enhances the ability to model irregular target shapes, and the channel attention mechanism further optimizes feature selectivity and weight assignment. This design allows the network to maintain excellent lightweight performance while maintaining high performance, making it ideal for applications in resource-limited devices and complex scenarios.

### 4.1. Multi-Scale Attention Feature Extraction Backbone

**Figure 4:**
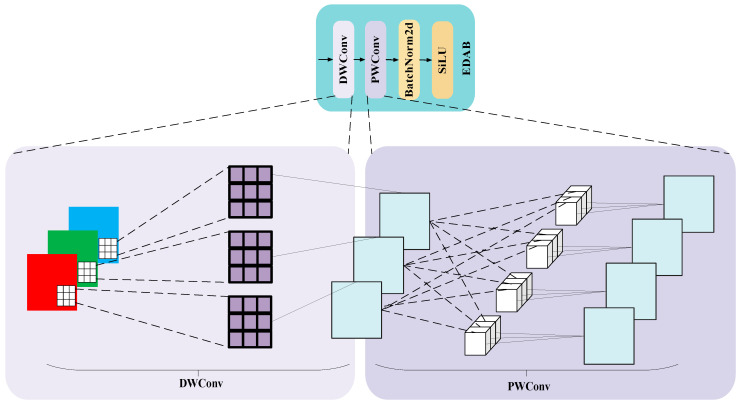
EDAB structural diagram.

Multi-scale feature extraction is the key to achieving efficient feature characterization for deep learning models in target detection and segmentation tasks. In this study, a novel network structure combining EDAB and MSCA is designed in the backbone part. Assuming an input image $X\in {\mathbb{R}}^{B\times C\times H\times W}$, each channel of the input is firstly subjected to an independent convolution operation by a 3 × 3 convolution kernel, followed by fusion of the inter-channel information using a 1 × 1 convolution kernel, and the number of output channels is Cout. Then, the feature distribution is adjusted using the BN, and finally, an SiLU activation function is applied to further extract the more complex features. The structure is shown in Figure 4.

**Figure 5:**
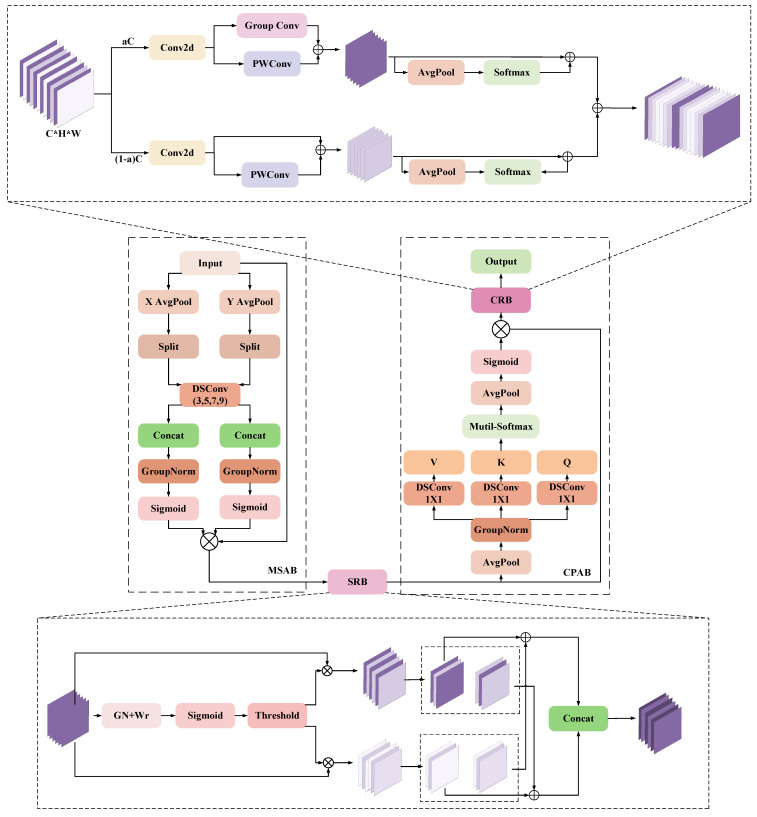
MSCA structural diagram.

The output of the above-mentioned preliminary feature extraction module is fed into the MSCA, and the structure of the unit is shown in Figure 5. It consists of multisemantic spatial attention block (MSAB), spatial reconstruction block (SRB), progressive channel attention block (CPAB), and channel reassembly block (CRB), each part is closely connected with each other and gradually strengthens the ability to express features.

MSAB is the first part of MSCA, which is mainly responsible for enhancing the expression ability of input features from different spatial directions, providing fine spatial context information, and providing high-quality initial input for subsequent modules to optimize feature structure. Specifically, the input feature $X\in {\mathbb{R}}^{B\times C\times H\times W}$ is pooled globally along the X and Y directions to generate a 1D sequence in the width and height directions, representing *X_H_* and *X_W_*, respectively and then applying grouping operations to *X_H_* and *X_W_* to extract the sub-features of different groups as follows:

(1) $$Split(X_{H},i)=X_{H}[:,(i-1)\cdot m:i\cdot m,:],i\in [1,K]$$

(2) $$Split(X_{W},i)=X_{W}[:,(i-1)\cdot m:i\cdot m,:],i\in [1,K]$$

where $m=\frac{C}{K}$ denotes the number of channels in each group, *K* is the number of groups, and (i−1)·m:i·m represents the selected channel range.

Next, a depth separable convolution operation is applied to each sub-feature $X_{H}^{\left(i\right)},X_{W}^{\left(i\right)}$ (with convolution kernel sizes of 3, 5, 7, 9), and they are spliced in height and width dimensions. Normalization is then performed by group normalization, and directional attention weights are generated by a gating mechanism (Sigmoid), as follows:

(3) $$Attn_{H}=Sigmoid(GN^{K}(Concat({\tilde{X}}_{H}^{\left(1\right)},{\tilde{X}}_{H}^{\left(2\right)},{\tilde{X}}_{H}^{\left(3\right)}\ldots \ldots {\tilde{X}}_{H}^{\left(K\right)})))$$

(4) $$Attn_{W}=Sigmoid(GN^{K}(Concat({\tilde{X}}_{W}^{\left(1\right)},{\tilde{X}}_{W}^{\left(2\right)},{\tilde{X}}_{W}^{\left(3\right)}\ldots \ldots {\tilde{X}}_{W}^{\left(K\right)})))$$

where Concat denotes the splicing feature group along the channel dimension, and *GN^K^* represents group normalization.

Finally, the multi-semantic space features are weighted to the original input feature *X* to form the enhanced feature, as follows:

(5) $$MSA(X)=Attn_{H}⊗X+Attn_{W}⊗X$$

where ⊗ denotes element-by-element multiplication, weighting the original feature map with attention weights.

SRB is the second part of MSCA that directly receives the spatial features optimized by MSAB and further eliminates redundant information in spatial dimension. SRB provides more efficient feature input for channel optimization by evaluating the importance of different channels in the input features and extracting spatially significant information. The specific implementation is to normalize the feature $X^{\prime }=MSA(X)$ output from MSAB, as follows:

(6) $$X_{out}=GN(X^{\prime })=\frac{X^{\prime }-\mu }{\sqrt{\sigma^{2}+\varepsilon }}\cdot \gamma +\beta$$

where μ=Mean(X,axis=[H,W]) denotes the mean of the feature map in the spatial dimension, $\sigma^{2}=Var(X,axis=[H,W])$ denotes the variance of the feature map in the spatial dimension, ε denotes a small constant for avoiding divide-by-zero errors, and γ,β denotes the corresponding learnable scaling and offset parameters for each channel.

The importance of each channel is then evaluated using the γ parameter of the *GN* output, the weight value is obtained, and the final channel selection weight *W* is generated by the Sigmoid function, as follows:

(7) $$W_{\gamma }[i]=\frac{\gamma [i]}{\sum_{j=1}^{C}\gamma [j]},W=Sigmoid(W_{\gamma }\cdot X_{out})$$

The weight *W* divides the feature map into valid features $X_{1}^{w}$ containing semantic information and cluttered features $X_{2}^{w}$ containing redundant information, as follows:

(8) $$X_{1}^{w}=W_{1}⊗X,X_{2}^{w}=W_{2}⊗X$$

where *W*_1_ denotes that the weights in *W* that are greater than the threshold (set to 0.5) are set to 1 and the rest to 0, and *W*_2_ (1 − *W*_1_) denotes the remaining redundant weights.

The final spatial reconstruction features are finally generated by weighted summation and stitching operations, as follows:

(9) $$\begin{array}{l} X_{11}^{w}=W_{1}⊗X_{1}^{w},X_{22}^{w}=W_{2}⊗X_{2}^{w}\\ X_{12}^{w}=W_{1}⊗X_{2}^{w},X_{21}^{w}=W_{2}⊗X_{1}^{w} \end{array}$$

(10) $$X^{w1}=X_{11}^{w}+X_{22}^{w},X^{w2}=X_{21}^{w}+X_{12}^{w}$$

(11) $$X^{w}=Concat(X^{w1},X^{w2})$$

CPAB is the third part of MSCA, which is closely connected with SRB, and further optimizes the feature expression on the channel dimension through the lightweight attention mechanism, providing multi-dimensional global contextual information for the subsequent modules. The output feature *X_P_* of SRB is first compressed in spatial dimensions by a global pooling operation, a step that preserves global semantic information and significantly reduces computational complexity. Then, a depth-separable convolution is used to generate the query Q, key K, and value V. The original features are mapped to three different feature spaces, and a weight matrix is generated through a self-attention mechanism, which is used to weight the value feature V to generate a new channel feature *X_attn_*. Finally, the channel-attention feature *X_attn_* is further optimized through global pooling and normalization, and fused with the original input features, as follows:

(12) $$Q=W_{Q}*X_{p},K=W_{K}*X_{p},V=W_{V}*X_{p}$$

(13) $$A=Softmax(\frac{Q\cdot K^{T}}{\sqrt{C}}),\;X_{attn}=A\cdot V$$

(14) $$X_{O}=X_{P}⊗Sigmoid(GN(GlobalPool(X_{attn})))$$

where $W_{Q},W_{K},W_{V}\in {\mathbb{R}}^{C\times C}$ denotes the learnable projection matrix, * denotes the convolution operation, $A\in {\mathbb{R}}^{C\times C}$ denotes the inter-channel attention weight matrix, $\sqrt{C}$ denotes the normalization factor, and *X_O_* denotes the augmented output features.

CRB is the final part of MSCA, which receives the CPAB-optimized features and further refines the feature representation in the channel dimension. The final channel optimized output is provided through feature decomposition, group convolution, and fusion strategies. The output features *X_O_* from CPAB are first decomposed along the channel dimension into an upper channel part *X_up_* and a lower channel part *X_low_*. For *X_up_*, multi-level features are extracted using Group Conv and PWConv, and for *X_low_*, fine-grained information is preserved using PWConv and Concat operations. Finally, the upper and lower channel features are fused by the weighting coefficients generated by Softmax, as follows:

(15) $$X_{up}=W_{split}^{up}*X^{w},\;X_{low}=W_{split}^{low}*X^{w}$$

(16) $$Y_{1}=W_{G}*X_{up}+W_{P}*X_{up}$$

(17) $$Y_{2}=W_{P-low}*X_{low}\cup X_{low}$$

(18) $$\beta_{1},\beta_{2}=Softmax(GlobalPool(Y_{1}),GlobalPool(Y_{2}))$$

(19) $$Y=\beta_{1}\cdot Y_{1}+\beta_{2}\cdot Y_{2}$$

where $W_{split}^{up}\in {\mathbb{R}}^{\alpha C\times C},W_{split}^{low}\in {\mathbb{R}}^{(1-\alpha )C\times C}$ is a learnable 1 × 1 convolutional kernel, $\alpha \in \left(0,1\right)$ denotes the channel division ratio, $\beta_{1},\;\beta_{2}\in [0,1]$ denotes the fusion weights of the upper and lower channels, and Y denotes the optimized channel information.

### 4.2. Dual-Stream Feature Fusion Module

**Figure 6:**
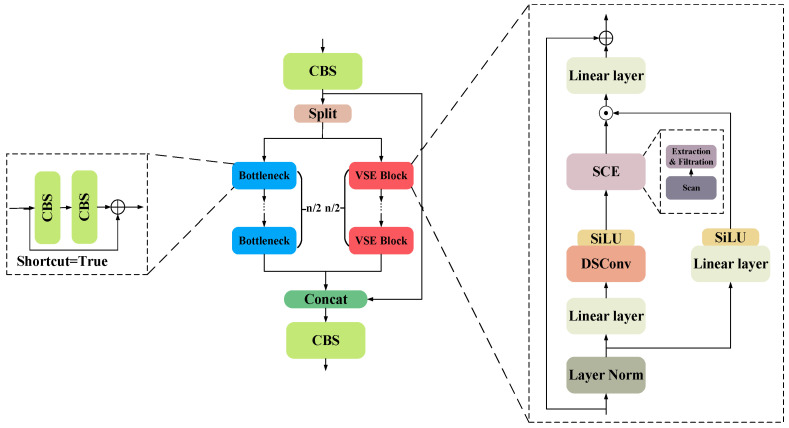
DSFFM structural diagram.

The direct splicing of encoder and decoder features often triggers a semantic mismatch problem, which affects the model performance. To solve this problem, this paper designs a dual-stream feature fusion enhancement module, which improves the expression of features and enhances the context consistency through a multi-path feature optimization mechanism. The structure is shown in Figure 6.

Initial fusion features are first generated by CBS processing. Subsequently, it is divided into two branches, the trunk path and the context path, which are used to capture local detail features and global context information, respectively. The backbone path gradually extracts local features through multiple bottleneck blocks (containing CBS and residual connections). The context path gradually extracts global features through multiple VSE blocks.

**Figure 7:**
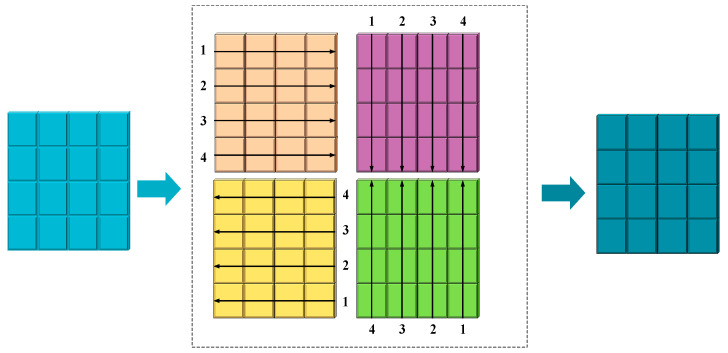
Schematic of the four different scanning processes. The markers 1–4 adjacent to each color indicate the four distinct scanning directions.

The context path is further divided into the following steps: The input features are normalized by layers and then divided into two branches; in the first branch, the input passes through the linear layer and the SiLU activation function in turn. In the second branch, the input first passes through the linear layer, depth separable convolution, and SiLU activation function in turn, and then, it feeds into the SCE module (which contains three parts: scanning, extraction, and filtering), which takes four different directions of scanning to unfold into a 1D sequence. The image scanning process is shown in Figure 7.

The features are then extracted and filtered using linear transformation and recursive computation, combined with exponential operations and feature decomposition mechanisms to distinguish between relevant and irrelevant information, and the input features are mapped into the following three feature subspaces by linear mapping:

(20) ΔA, B, C=Linear(x), Linear(x), Linear(x)

where $x\in {\mathbb{R}}^{B\times L\times D}$: *B*, *L*, *D* denote the batch size, sequence length, and feature dimension, respectively, Δ*A*, *B*, *C* denote the feature components generated by linear transformation, and Linear(·) denotes the linear mapping.

Secondly, in order to capture the nonlinear correlation of the features and efficiently model the contextual relationships in the sequence features, exponential operations and matrix decomposition are performed on the generated features Δ*A*, Δ*B*, and the recursive mechanism is used to update the hidden states, as follows:

(21) $$\bar{A}=exp(\Delta A),\;B={\left(\Delta A\right)}^{-1}\cdot (exp(\Delta A)-I)\cdot \Delta B$$

(22) $$h_{t}=\bar{A}h_{t-1}+Bx_{t}$$

where *exp*(Δ*A*) is an element-by-element exponential operation, *I* denotes the unit matrix, *h_t_* denotes the hidden state feature at the current moment, *h_t_*_−1_ denotes the hidden state feature at the previous moment, and *x_t_* denotes the input feature at the current moment.

Next, it combines the hidden state feature *h_t_* with the current input feature *x_t_* and stitches all the moments to obtain the complete output feature, as follows:

(23) $$y_{t}=Ch_{t}+Dx_{t}$$

(24) $$y=[y_{1},y_{2},y_{t},\ldots \ldots ,y_{L}]$$

where *C* denotes the weights for adjusting the hidden state features, *D* denotes the redundant information used to filter the current input features, and *y* denotes the optimized context information.

Finally, the first and second branch outputs are merged and mixed by element-by-element multiplication, and the outputs of the VSE Block are generated by residual concatenation. The output features of trunk path and context path are merged again with the features processed by CBS through Concat operation and are further optimized by CBS to generate the final output features.

### 4.3. Adaptive Context-Aware Detection and Segmentation Head

**Figure 8:**
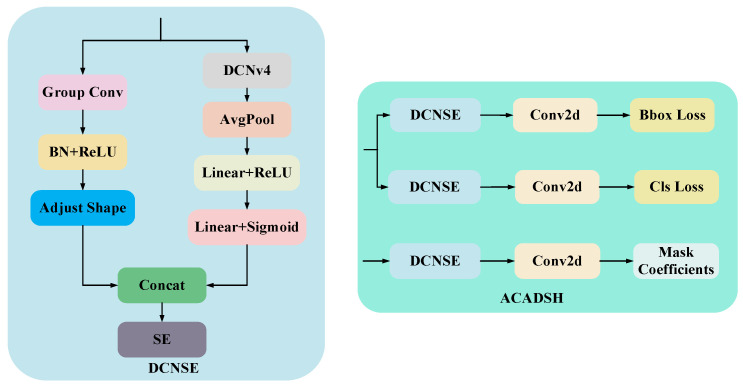
ACADSH structural diagram.

In the task of target detection and instance segmentation, the detection head and segmentation head are the key output parts in the whole network. In order to improve the detection accuracy and segmentation performance while considering the computational efficiency, this paper designs a unified detection and segmentation head module based on deformable convolution. This module can simultaneously handle bounding box prediction (BBox) and category prediction (Cls) for target detection and mask coefficient prediction (mask coefficients) for instance segmentation, and its structure is shown in Figure 8.

It is mainly composed of the following three parts: deformable convolution with squeeze and excitation (DCNSE), Conv2D, and loss calculation unit. The end-to-end outputs of detection and segmentation are realized uniformly through the multi-task structure design.

Among them, DCNSE is the core of the detection and segmentation head, which aims to enhance the modeling capability of target shape and local features through DCNv4 and, at the same time, aims to adaptively adjust the importance of different channel features by combining with the channel attention mechanism (Squeeze-and-Excitation, SE). On the one hand, GroupConv, BN, and ReLU activation functions are used to reduce the number of parameters and improve the training stability and nonlinear representation. On the other hand, the local and geometric deformation features of the input features are extracted by DCNv4, and then the output features are subjected to global average pooling (AvgPool), which compresses the spatial dimensions and retains only the global statistical information of the channels. Then, the pooled features are nonlinearly transformed by linear mapping with the ReLU activation function to enhance the expressive capability; subsequently, the channel weights are generated by a second linear layer with the Sigmoid activation function to complete the adaptive weighting of the importance of different channels.

The output of DCNSE enters two sub-task branches, detection head and segmentation head. The detection branch is responsible for predicting bounding box regression and category classification. The segmentation branch is used to predict the mask coefficients.

## 5. Experiments and Results

### 5.1. Data Acquisition and Production

**Figure 9:**
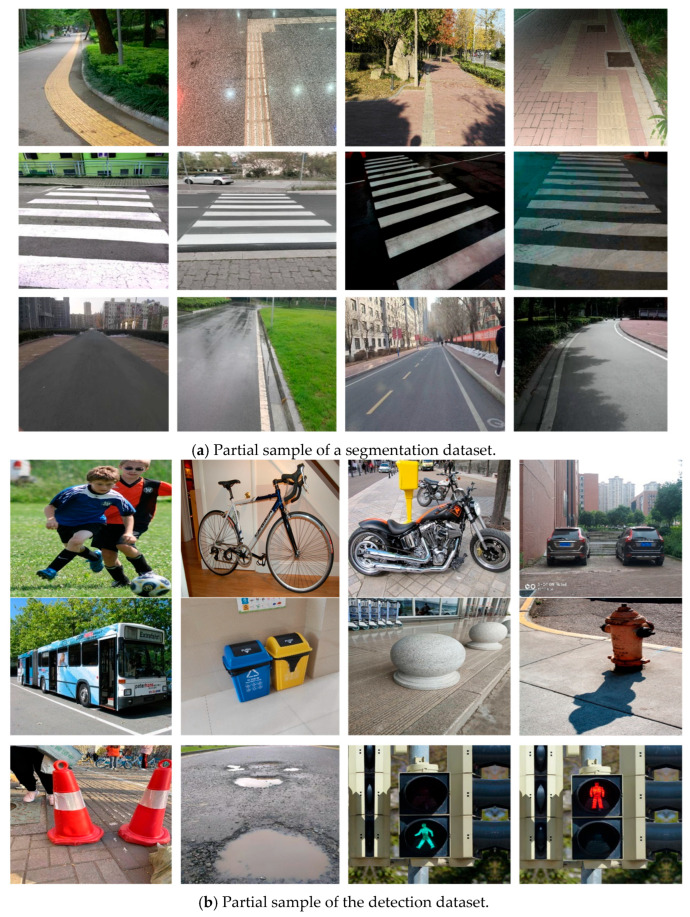
Partial sample of the dataset: (**a**) Segmentation dataset; (**b**) Detection dataset.

The road area where blind people walk is extended to three categories of blind roads, pedestrian crossings, and ordinary roads as the dataset needed for segmentation, a sample of which is shown in Figure 9a. The blind road data originate from the open-source TP-Dataset [16], containing 1391 images of various typical scenes, including campuses, streets, railway stations, bus stations, underground areas, communities, and hospitals. Pedestrian crossing data, comprising 1100 images, were sourced from the Kaggle platform. Ordinary road data were taken from the All-Weather Road Image Segmentation dataset (UAS) [17], which includes four types of weather conditions, including dusk, night, rain, and bright sunshine, totaling 6380 images. The obstacle detection dataset, on the other hand, is taken from the dataset website (Roboflow), covering 12 categories such as pedestrians, bicycles, motorbikes, cars, buses, bins, stone piers, fire hydrants, conical barrels, puddles, green lights at pedestrian intersections, and red lights at pedestrian intersections, totaling 12,300 images, and a sample of the dataset is shown in Figure 9b.

To ensure that the data categories are balanced, we randomly selected 1500 images from the general road dataset as part of the segmentation dataset. Since the category labels of the segmentation dataset are all binarized mask images, we processed them using OpenCV’s color and edge detection techniques to convert them into the form of coordinates required by the segmentation network. In the obstacle detection dataset, we performed a comprehensive data cleaning operation to eliminate all images with mislabeling and omission to ensure the accuracy and quality of the data. Finally, the processed segmentation and detection dataset was divided into training, validation, and test sets in the ratio of 8:1:1 and fed into the model for training.

### 5.2. Image Processing

**Figure 10:**
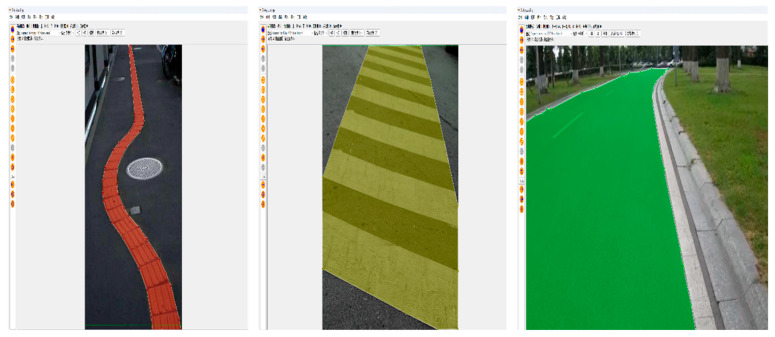
Dataset labeling process.

Due to the limited sample size in the segmented dataset, which can lead to poor model generalization, we collected road videos around the school campus. Images were extracted from these videos via frame extraction and combined with data sourced from major dataset repositories and web crawling. This merged dataset was then significantly expanded using data augmentation techniques, including rotation, scaling, translation, color transformation, and contrast adjustment. The total number of images reached 6660 (including 2250 images of blind roads, 2310 images of crosswalks, and 2100 images of ordinary roads), and finally the images were manually labeled using the semi-automatic labeling software X-Anylabeling (https://github.com/CVHub520/X-AnyLabeling/tree/main accessed on 5 July 2025), and the labeled dataset is shown in Figure 10.

### 5.3. Experimental Setup and Environment

The hardware and software configuration of the training environment for this experiment is shown in Table 2. The training uses stochastic gradient descent (SGD) and sets the initial learning rate to 0.01, the momentum factor to 0.937, the regularization factor to 0.0005, the input image size to 640 × 640 pixels, the number of batch processes (batch size) to 32, and the training cycles (epochs) to 200 rounds, and it automatically stops the training if there is no performance improvement in 50 cycles. In addition, we used the Mixup data enhancement technique (specifically, the two images were blended in proportion after data enhancement such as flipping, zooming, and color gamut changes) to improve the model performance. The training environment is configured as shown below.

For the experimental inference part, Jetson Nano introduced above is used as the inference platform. It has low power consumption and strong computational capability, which is suitable for deep learning applications in edge devices. In order to comprehensively assess the effectiveness of the improved algorithm in this paper, the following evaluation metrics were used: model scale size (MB), computation amount (GFLOPs), number of parameters (M), mean average precision (mAP), mask mean average precision (mAP_mask_), and frame rate (FPS). The model scale size, computation volume, and number of parameters were used to measure the storage and computation requirements of the model, while mAP, mAP_mask_, and FPS were used to evaluate the detection accuracy and real-time performance of the model.

### 5.4. Ablation Experiments

In order to verify the effectiveness of the improved network and enhance the robustness of the model, the ablation experiments were designed by dividing the improved method into the following three parts: firstly, adding MSAFEB; secondly, adding DSFFM; and thirdly, adding ACADSH. The results of the ablation experiments are shown in Table 3 and Table 4.

**Figure 11:**
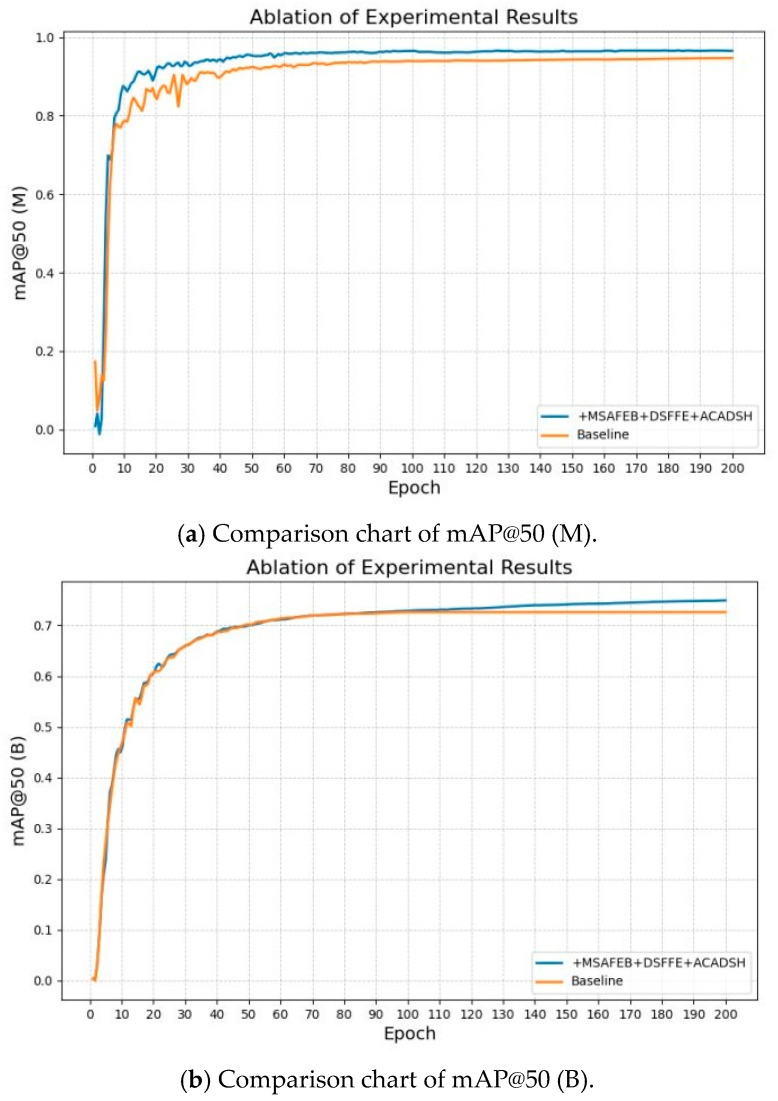
Comparison of experimental results: (**a**) Comparison chart of mAP@50 (M); (**b**) Comparison chart of mAP@50 (B).

As seen in Table 4, in the road segmentation task, using MSAFEB, DSFFM, and ACADSH, respectively, the mask accuracy was improved by 1.1, 1.4, and 0.9 percentage points, and compared with the original network, the improved network model improved by 2.7 percentage points. A comparison of the results before and after the improvement is shown in Figure 11a, from which the improved model can reduce some training process fluctuations during the training process and converge faster to reach a stable value. In addition, the table shows that the improved module combination reduced the model size by 7.3%, the number of parameters by 5.2%, and the GFLOP by 9.8%. The frame rate was kept above 95 frames/s, which has strong real-time performance and can meet the demands of many real-world scenarios. Overall, the improved module effectively enhanced the comprehensive performance of the segmentation task and achieved a good balance between accuracy and efficiency.

As seen in Table 4, the detection accuracy (mAP) was improved by 1.5, 1.9, and 0.2 percentage points in the obstacle detection task using MSAFEB, DSFFM, and ACADSH, respectively. Compared with the baseline network, the improved network model had an overall improvement of 3.1 percentage points, and a comparison of the results before and after the improvement is shown in Figure 11b. In addition, as can be seen from the table, the improved module combination reduced the model size by 6.6%, the number of parameters by 7.1%, and the GFLOP by 7.3%. Meanwhile, the frame rate was kept above 90 frames/s. The improved model not only significantly reduced the computational complexity, but it also considered the efficient inference speed, which fully met the real-time and resource-friendly requirements of the detection model in practical applications.

### 5.5. Comparative Experiments

To further validate the superiority of the proposed algorithm and demonstrate that its improvements genuinely enhance model performance, we conducted comparative experiments under identical datasets and experimental conditions. The four typical example segmentation networks, yolov9c-seg, yolo11s-seg, SAM-2, and Mask-RT-DETR, were used to compare the road segmentation task network, and the comparison of evaluation indexes of the experimental results is shown in Table 5. Meanwhile, the four latest target detection networks, yolov10n, yolo11, Grounding DINO, and RT-DETR, were used to compare the obstacle detection network, and the evaluation index comparison of the experimental results is shown in Table 6.

**Figure 12:**
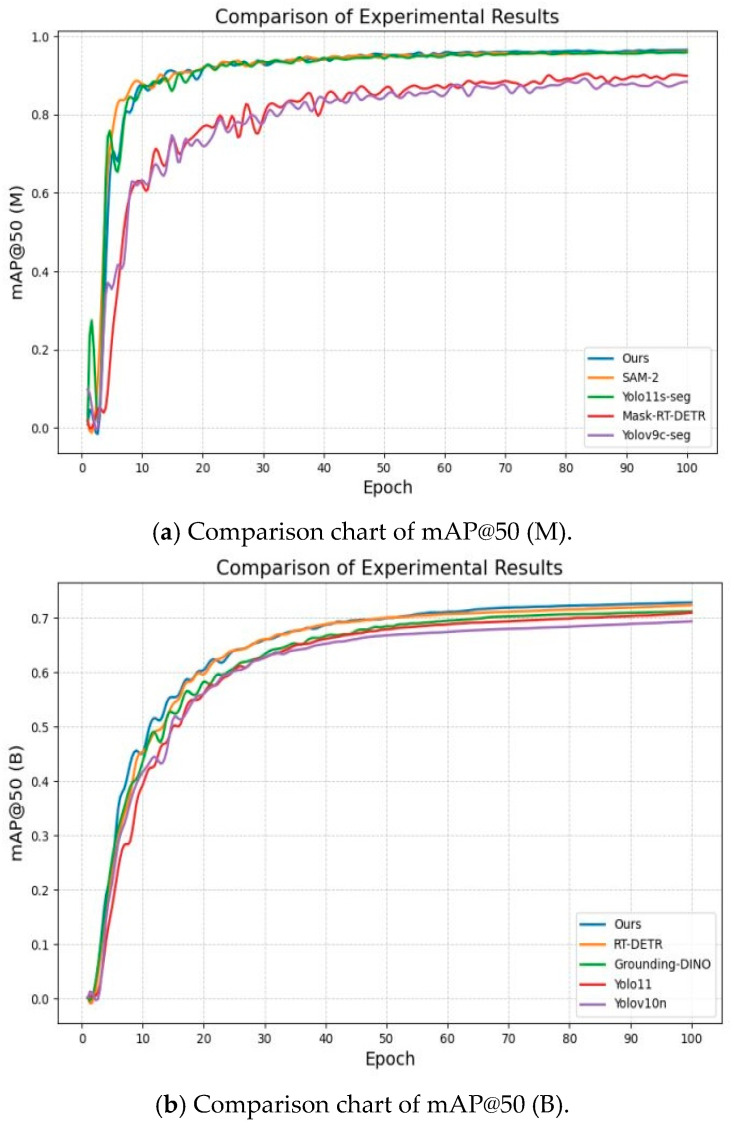
Comparison of experimental results: (**a**) Comparison chart of mAP@50 (M); (**b**) Comparison chart of mAP@50 (B).

The data in Table 5 show that the model proposed in this paper exhibited excellent performance in the road segmentation task, reaching an mAP of 0.979, which is the highest accuracy in the table, while maintaining a real-time performance of 98 FPS, which considers the balance between accuracy and efficiency. In addition, the model size was only 5.1 MB, the number of parameters was 2.69 M, and the computational complexity was 9.8 GFLOPs, which significantly outperformed the other comparative methods in terms of lightness and efficiency, and the results of the algorithm comparison are shown in Figure 12a. This shows that the model proposed in this paper greatly reduces the computational cost while improving the segmentation accuracy, and it can better adapt to the actual scene requirements, which fully verifies the advancement and effectiveness of its design.

As shown in Table 6 and Figure 12b, the proposed model achieved a state-of-the-art performance in obstacle detection, achieving the highest accuracy (75.7% mAP) while maintaining real-time inference (98 FPS). In addition, its lightweight architecture (model size was only 4.7 MB, parameter was 2.47 M, and computational complexity was 6.1 GFLOPs). Together, these results demonstrate the model’s good balance between accuracy, speed, and resource efficiency, validating its great potential for real-world deployment in resource-constrained scenarios.

### 5.6. Algorithm Results Visualization and Analysis

**Figure 13:**
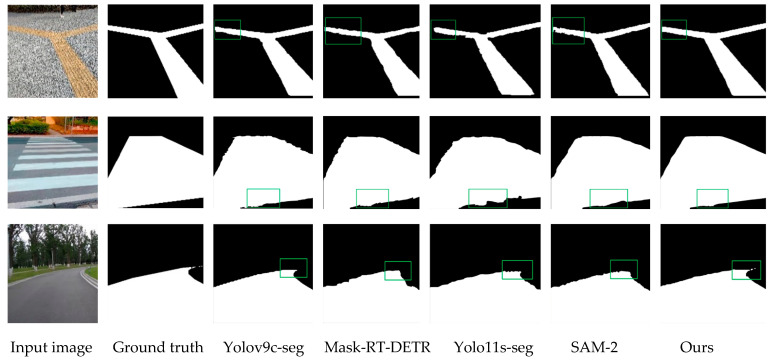
Comparison of the effects of different algorithms.

To visually demonstrate the superior performance of the improved algorithm in this paper for road segmentation, we selected several images from the dataset for visualization, as shown in Figure 13. The boundary segmentation in the green box and the overall segmentation area of the improved algorithm in this paper are better than those of the other algorithms.

**Figure 14:**
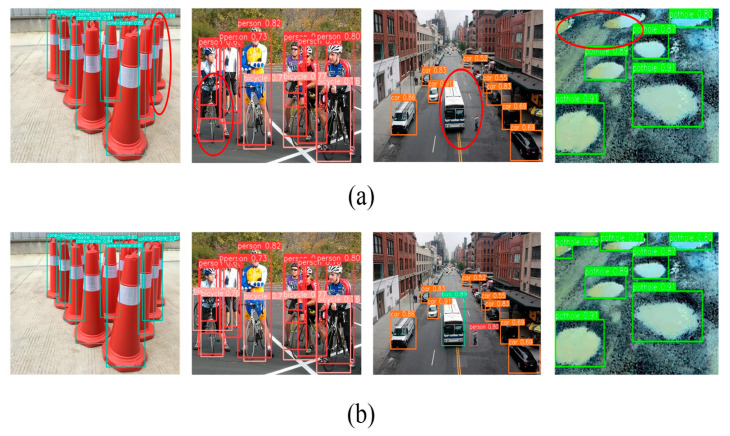
Performance comparison between the baseline (YOLOv8n) and our improved model.

To demonstrate the enhancement of our obstacle detection model specifically for challenging scenarios encountered by the visually impaired, we selected four complex travel environment images for visualization (Figure 14).

The baseline YOLOv8n model exhibited false negatives in detecting small and occluded obstacles (highlighted in red circles) in Figure 14a,b. In contrast, our proposed model showed significant improvements in detecting these critical targets, effectively mitigating false negatives and misdetections during blind navigation.

### 5.7. Real-World Scenario Test Visualization and Analysis

**Figure 15:**
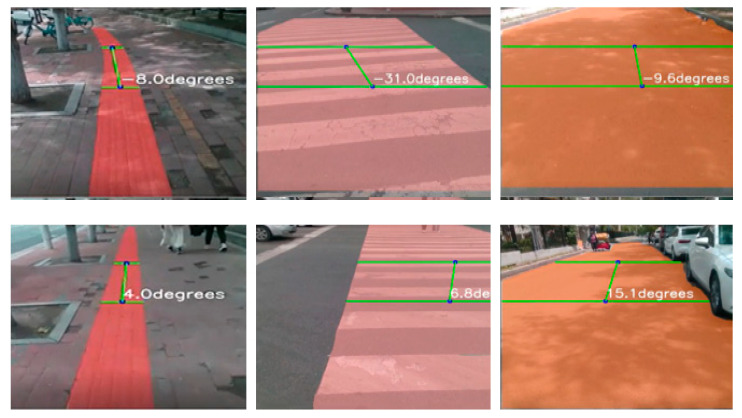
Direction guidance design.

The design of the directional guidance system for the blind is as follows: The system first uses a segmentation algorithm to identify the road area and converts the recognition results into a binary image to distinguish between walkable and non-walkable areas. Then, it establishes two baselines to determine the boundaries of the white area (walkable area), obtains the central coordinates of the two baselines, and calculates the slope K1 of the line connecting the center point of the road (K1 reflects the direction of the current road extension relative to the user’s front). If K1 < −10°, it indicates that the road extends towards the left front, and the system will prompt the user to adjust to the left front through servo vibrations; if −10° < K1 < 10°, it indicates that the road is generally extending directly in front of the user, and at this time, the system does not issue a directional adjustment prompt; if K1 > 10°, it indicates that the road extends towards the right front, and the system will prompt the user to adjust to the right front through servo vibrations. The corresponding road extension directions for different K1 values are shown in Figure 15.

**Figure 16:**
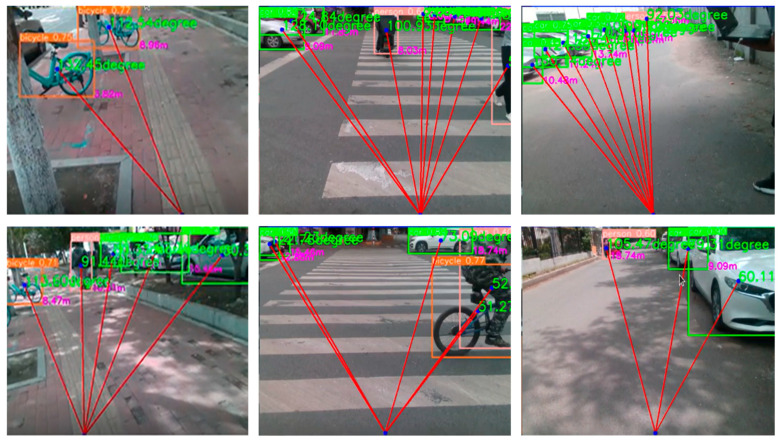
Obstacle avoidance strategy design.

The obstacle detection and obstacle avoidance strategy is designed as follows: the system first uses the D435i depth camera to obtain the distance information of the front environment, and when the detected obstacle distance is less than the preset safety threshold, the system will further analyze the position of the obstacle. By connecting the bottom center point of the road split area with the center point of the target obstacle detection bounding box, the slope K2 of the calculated line can be determined (K2 represents the orientation of the obstacle relative to the center line of the road). If 75° < K2 < 90°, then the obstacle is in front of the user’s right, and if it is 90° < K2 < 105°, it means that the obstacle is in front of the user’s left. The system will issue corresponding obstacle avoidance prompts to the user according to the specific orientation (left front/right front) and distance of the obstacle (if there is an obstacle in front of the left, the left ear servo will vibrate, and the user will avoid it to the right; if there is an obstacle in front of the right, the right ear servo will vibrate, and the user will avoid it to the left). The orientation of the obstacle corresponding to different K2 values is shown in Figure 16.

**Figure 17:**
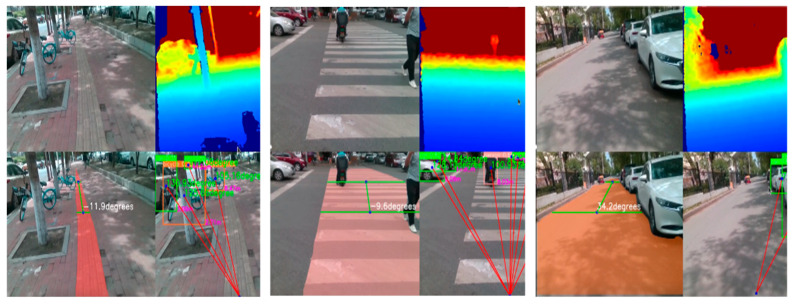
Test results in different scenarios.

Finally, we deployed the trained model on an NVIDIA Jetson Nano edge device, captured color and depth images simultaneously with the D435i depth camera, and performed road segmentation (identifying walkable areas, calculating road center offsets) and obstacle detection (identifying obstacles and obtaining information about their distance and angle) in parallel. In the multi-scenario field test (Figure 17), the system showed a good segmentation effect under various road types, and it could effectively detect all kinds of static or dynamic obstacles encountered by blind people, even in a partially occluded state. Figure 17 shows the typical test results, the original image on the top left, the depth image on the top right, the road segmentation post-processing effect on the bottom left, and the obstacle detection post-processing effect on the bottom right.

## 6. Conclusions

In this paper, we design and implement a set of intelligent travel assistive devices based on deep learning and edge computing to address the challenges faced by the BVI people in the process of traveling. The device combines an advanced hardware platform and optimized deep learning algorithms, aiming to enhance the safety and autonomy of BVI people in complex environments. The device uses NVIDIA Jetson Nano as the edge computing platform and is equipped with a D435i depth camera for environment sensing, thus realizing real-time depth information capture of the surrounding environment. With lightweight detection and segmentation algorithms, this device can efficiently perform obstacle detection and road segmentation.

In terms of hardware design, the device adopts a compact and efficient modular structure, integrating key components such as the main control unit, the environment sensing unit, and the feedback unit, ensuring the portability and practical applicability of the device. As for the software algorithm, the device can achieve smooth real-time operation on resource-constrained embedded systems through careful lightweight improvement. The experimental results show that the device exhibits excellent performance in both road segmentation and obstacle detection tasks; the mAP_mask_ of the road segmentation task reaches 97.9%, and the mAP of the obstacle detection task reaches 75.7%, and the device maintains a high frame rate (FPS) while maintaining high accuracy, which fully meets the real-time demand.

Compared to existing technologies, this device not only excels in accuracy but also achieves lightweight design and high performance, holding significant commercial value and broad application prospects. Real-world test results across multiple scenarios validate its effectiveness and robustness. Through its vibration feedback mechanism, the device provides real-time navigation prompts and obstacle warnings for BVI people, which could potentially help them walk outdoors and avoid obstacles more safely.

## Figures and Tables

**Table 1:** Jetson Nano hardware configuration table.

NVIDIA Jetson Nano	
GPU	128-core Maxwell
CPU	Quad-core ARM A57 @ 1.43 GHZ
Memory	4 GB 64-bit LPDDR4 25.6 GB/s
Storage	microSD (not included)
Video encoding	4K @ 30|4× 1080p @ 30|9× 720p @ 30 [H.264/H.265)
Video decoding	4K @ 60|2× 4K @ 30|8× 1080p @ 30 [H.264/H.265)
Camera	1× MIPI CS1-2 DPHY lanes
Connectivity	Gigabit Ethernet, M.2 Key E
Display	HDMI 2.0 and eDP 1.4
USB interface	4× USB 3.0, USB 2.0 Micro-B
Other	GPIO, 2C, 2S, SPI. UART
Mechanical part	69 mm × 45 mm, 260-pin edge connector

**Table 2:** Experimental platform configuration.

Project	Configure
Operating System	Ubuntu20.04
Graphics Card	GeForce RTX 3060 (12 GB)
CUDA Version	11.8
Python	3.8.16
Deep Learning Framework	Pytorch1.13.1

**Table 3:** Road segmentation ablation experiment.

Model	Model Size	Parameters	GFLOPs	mAP_mask_	FPS
Baseline	6.8	3.26	12.1	0.952	67
+MSAFEB	5.6	2.74	10.2	0.963	94
+DSFFM	6.4	3.18	11.8	0.966	85
+ACADSH	5.9	2.96	10.5	0.961	88
+MSAFEB+DSFFM	5.9	2.84	10.9	0.971	95
+MSAFEB+ACADSH	5.4	2.79	10.3	0.972	94
+DSFFH+ACADSH	6.1	3.09	11.2	0.971	90
+MSAFEB+DSFFE+ACADSH	5.1	2.69	9.8	0.979	98

**Table 4:** Obstacle detection ablation experiment.

Model	Model Size	Parameters	GFLOPs	mAP	FPS
Baseline	6.3	3.01	8.2	0.726	73
+MSAFEB	5.7	2.86	7.9	0.741	93
+DSFFM	6.2	2.97	6.7	0.732	87
+ACADSH	5.9	2.91	7.3	0.728	85
+MSAFEB+DSFFM	5.4	2.64	6.5	0.749	97
+MSAFEB+ACADSH	5.8	2.63	7.1	0.743	95
+DSFFH+ACADSH	6.0	2.81	6.9	0.741	92
+MSAFEB+DSFFE+ACADSH	5.2	2.47	6.1	0.757	98

**Table 5:** Comparison of road segmentation algorithms.

Model	Model Size	Parameters	GFLOPs	mAP_mask_	FPS
Yolov9c-seg [18]	53.9	27.89	159.4	0.901	89
Yolo11s-seg [19]	19.7	10.1	35.3	0.971	91
SAM-2 [20]	74.4	33.5	79.8	0.977	76
Mask-RT-DETR [21]	72.9	35.3	81.5	0.913	83
Ours	5.1	2.69	9.8	0.979	98

**Table 6:** Comparison of obstacle detection algorithms.

Model	Model Size	Parameters	GFLOPs	mAP	FPS
Yolov10n [22]	5.2	2.6	7.7	0.691	65
Yolo11 [19]	6.5	3.84	6.7	0.722	68
Grounding DINO [23]	89.9	42.1	107.7	0.726	79
RT-DETR [21]	63.4	27.2	73.4	0.751	85
Ours	4.7	2.47	6.1	0.757	98

## Data Availability

The author will provide the original data supporting the conclusions of this article as required.
